# Early health outcome and 10-year survival in patients undergoing redo coronary surgery with or without cardiopulmonary bypass: a propensity score-matched analysis

**DOI:** 10.1093/ejcts/ezx137

**Published:** 2017-05-15

**Authors:** Vito D Bruno, Mustafa Zakkar, Filippo Rapetto, Asif Rathore, Roberto Marsico, Pierpaolo Chivasso, Raimondo Ascione

**Affiliations:** School of Clinical Sciences, Bristol Heart Institute, University of Bristol, Bristol, UK

**Keywords:** Coronary surgery, Reoperation, Off-pump technique, Survival, Cardio-pulmonary bypass

## Abstract

**OBJECTIVES:**

To investigate the in-hospital health outcome and 10-year survival in patients undergoing redo coronary surgery with (redo-CABG) or without (redo-OPCAB) cardiopulmonary bypass.

**METHODS:**

A total of 349 redo coronary surgery patients were identified from our registry. Of these, 143 redo-OPCAB patients (40.97%) were compared with 206 redo-CABG patients. To minimize the bias, we also conducted propensity score matching. In Matched Analysis A, 111 redo-OPCAB patients with any type of primary cardiac operation were compared with 111 redo-CABG cases. In Matched Analysis B, 84 redo-OPCAB patients with isolated coronary surgery as their primary operation were compared with 84 redo-CABG patients. We assessed for all 3 analyses a composite of in-hospital mortality, acute kidney injury, stroke and severe low cardiac output requiring intra-aortic balloon pump. In addition, we assessed 1-, 5-, and 10-year survival.

**RESULTS:**

In the unmatched analysis, redo-CABG was associated with higher usage of intra-aortic balloon pump (10 vs 3%, *P* = 0.01) and composite compared with redo-OPCAB (25 vs 16%, *P* = 0.06) and similar 10-year survival (67.2 vs 68.5%, log-rank test: *P* = 0.78). Matched Analysis A showed similar rates of composite (15 vs 21%, *P* = 0.25) and 10-year survival (65.1 vs 60.8%, log-rank test: *P* = 0.5). Matched Analysis B showed reduction of the composite (19 vs 8%, *P* = 0.04), less in-hospital mortality (5 vs 0%, *P* = 0.13), 4.5 times less need for intra-aortic balloon pump (2 vs 11%, *P* = 0.02) favouring redo-OPCAB and a similar 10-year survival (71.6 vs 71.7%, log-rank test: *P* = 0.61).

**CONCLUSIONS:**

Redo-OPCAB surgery is feasible, safe and effective with improved in-hospital outcome and similar 10-year survival compared to redo-CABG.

## BACKGROUND

Redo cardiac surgery represents 5–8% of the current surgical practice [1–4]. In particular, reoperative coronary artery bypass grafting (redo-CABG) remains challenging [1–4] with prolonged operation time, bleeding, poor access to coronary targets and risk of coronary microembolization leading to complications [2, 3]. Redo-CABG has declined over the last decade to 2.5–4% of all coronary procedures, probably due to improved platelet inhibition [2, 3], use of more arterial conduits or to the use of percutaneous coronary intervention stenting to treat occluded grafts or new stenosis [5]. Poor risk profile in these patients may trigger complications associated with prolonged cardiopulmonary bypass and cardioplegic arrest time [6]. Studies from North America, Japan and India have highlighted the use of off-pump CABG as an alternative to redo coronary surgery (redo-OPCAB) [1, 6, 7]. However, most of these studies are based on small patient cohorts, and none of these studies has evaluated the impact of redo-OPCAB on long-term survival. In this study, we assess the safety and efficacy of redo-OPCAB versus redo-CABG using a combined end-point of serious postoperative complications as well as 1-, 5-, and 10-year survival.

## MATERIALS AND METHODS

We undertook a retrospective analysis from our database registry. Data were prospectively collected, validated and stored by an independent team as part of the UK National Registry. The study protocol was in compliance with the local Institutional Clinical Audit Review Board, and patient consent was waived.

### Patient selection

**Figure 1:**
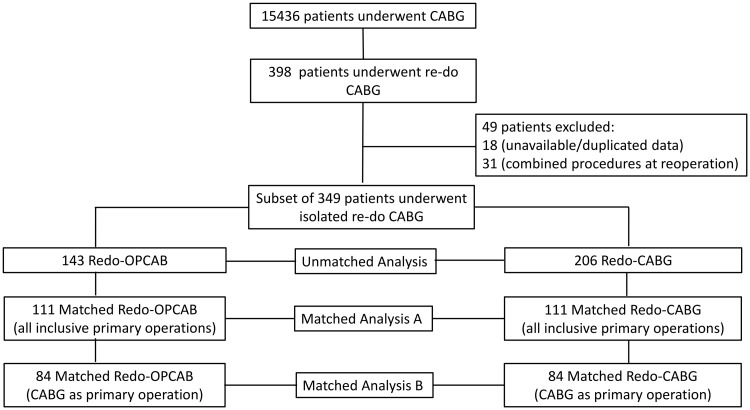
Diagram of patient’s selection.

Between May 1996 and January 2014, 15 436 patients underwent isolated coronary surgery. From these, we selected the study population as shown in Fig. 1. First, 349 patients were identified to have undergone any redo coronary surgery, with any primary cardiac procedure (isolated CABG, valve only or CABG plus valve/other). Of these, 143 (40.97%) received redo-OPCAB and represented the study group. The remaining 206 patients (59.03%) received redo-CABG and formed the control group. Next, we selected a subgroup of 273 of 349 redo patients with isolated coronary surgery only being the primary procedure. Of these, 100 patients (36.6%) underwent redo-OPCAB and 173 patients (63.4%) underwent redo-CABG.

### Data collection and clinical management

Baseline data included clinical characteristics, symptom status, past medical history, markers of renal function including serum creatinine (SCrea) and estimated glomerular filtration rate. Postoperative levels of SCrea were obtained from medical records and estimated glomerular filtration rate calculated accordingly [8]. Indication for surgery was based on coronary angiography. The use of redo-OPCAB was based on surgeons using predominantly OPCAB or CABG with none undertaking a mixed practice. The OPCAB technique used was as previously reported [9, 10] using pressure stabilizer and intracoronary shunts. Anaesthesia and perfusion techniques were also as previously reported [11–14].

Computed tomography angiography was used according to surgeon preference to ascertain the anatomical relation of the heart and grafts with the re-entry route. Left ventricular ejection fraction was derived from baseline echocardiogram or left ventriculogram. Our definition of reduced left ventricular ejection fraction was <50%. Intraoperative and postoperative data collection and clinical management were as previously reported [8]. Patients developing non-cardiac single organ renal, respiratory and/or neurological dysfunction/failure were treated till complete recovery and referred to a specialist centre for further management beyond the 30-days cut-off, if necessary.

### Outcome measures and definitions

The selected composite end-point included: in-hospital mortality, acute kidney injury (AKI), cerebrovascular accidents and severe low cardiac output (LCO), requiring intraoperative or postoperative intra-aortic balloon pump (IABP). Reopening for bleeding and length of hospital stay were also recorded. We also assessed 1-, 5- and 10-year survival. In-hospital mortality was defined as death by any cause occurred at any time before discharge from index hospitalization, regardless the length of hospital stay. The 30-day mortality was defined as death within 30 days from surgery even if occurring after discharge. Cerebrovascular was defined as any new postoperative stroke confirmed by computed tomography scan. AKI was defined according to the Risk, Injury, Failure, Loss, and End stage (RIFLE) criteria [14]. The highest postoperative creatinine value recorded was used to compare with baseline value, bearing in mind that RIFLE criteria require SCrea to increase by at least 50% from baseline value to be classified as AKI [14]. Severe LCO was defined as any intraoperative or postoperative LCO requiring the use of IABP. Late survival data were obtained from the UK National Health Service (NHS) tracing service to identify non-survivors and date of late any-cause death, with the latest data obtained in February 2015.

### Statistical analysis

Data are presented as mean ± one SD for continuous variables or as percentages for dichotomous variables. Continuous numerical variables were tested for normality using the Shapiro–Wilk test and then compared between groups with unpaired Student’s *t*-test if normally distributed or Mann–Whitney *U*-test if not normally distributed. In the case of dichotomous variables, Pearson’s *χ*^2^ or Fisher’s exact test were used as appropriate. Predictors for composite outcome were tested using a multivariable logistic regression model: a stepwise selective approach was conducted by backward and forward selection methods using Akaike information criterion as discrimination criterion between models. Event-free survival curves were compared between the groups by Kaplan–Meier method and subsequently compared with the log-rank test. To further adjust for patient selection and preoperative characteristics, we developed 2 propensity score-matched analyses including in the analysis all the baseline variables available. In Matched Analysis A, we included all the identified redo coronary surgery patients, regardless of the type of primary operation. In Matched Analysis B, we included only patients who had undergone isolated coronary surgery as their primary operation. In both cases, patients undergoing redo-OPCAB were matched (1:1) to the group undergoing redo-CABG by all the preoperative variables. Intraoperative variables were not included in the model as they occurred during the surgery. The nearest neighbour method was used with a caliper of 0.2 and the balance after matching was evaluated with standardized mean differences. After propensity score matching, variables were compared using paired Student’s *t*-test or paired Wilcoxon test for continuous variables and McNemar (for dichotomous variables) and *χ*^2^ test for ordinal categorical variables. A conditional logistic regression model was developed to evaluate the predictors of the composite outcome including the same variables used for the non-matched unconditional logistic regression plus the matching index. All tests were two-sided with the *α* level set at 0.05 for statistical significance. Clinical data were recorded and subsequently tabulated with Microsoft Excel (® Microsoft Corp, Redmond, WA, USA). The statistical analysis was computed using R version 3.0.2 [R Core Team (2014), R Foundation for Statistical Computing, Vienna, Austria]. The propensity score matching was computed with the package MatchIt [15].

## RESULTS

### Unmatched analysis

Baseline patient characteristics are presented in Table 1. The overall mean age was 66.6 ± 8.5 years and 53 patients (15%) were female. Patients in the redo-OPCAB group were on average 2 years older (67.8 ± 8.1 vs 65.7 ± 8.6 years, *P* = 0.02) and had twice the amount of peripheral vascular disease (27% vs 16%, *P* = 0.02). The average number of diseased coronary vessels was lower in the redo-OPCAB (2.3 ± 0.8 vs 2.6 ± 0.6, *P* < 0.01) reflecting the number of bypass grafts performed (2.3 ± 0.8 vs 2.6 ± 0.6, *P* < 0.01). Completeness of revascularization was similar at 76% vs 73% redo-OPCAB vs redo-CABG respectively (*P* = 0.9). Postoperative outcomes are shown in Table 2. The incidence of the composite end-point was higher in the redo-CABG group (25% vs 16%, *P* = 0.06). In-hospital mortality was 2.8 times higher in the redo-CABG group (6 vs 2%, *P* = 0.09). The other variables of the composite included: postoperative AKI (16 vs 13%, *P* = 0.43), stroke (1 vs 1%, *P* = 0.78) and severe LCO needing IABP (10 vs 3%, *P* =0.01), all redo-CABG vs redo-OPCAB respectively. Peak of postoperative SCrea was 134.7 ± 85.1 µmol/l and 126.04 ± 62.3 µmol/l for the redo-CABG and redo-OPCAB respectively (*P* = 0.30). Reopening for bleeding was higher in redo-CABG group (3 vs 1%, *P* =0.19), while hospital stay was 0.6 days longer (8.8 ± 6.9 vs 8.2 ± 6.2 days, *P* = 0.07) in the same group.

**Table 1:** Pre- and intraoperative characteristics

	Unmatched analysis			
	Overall (*n* = 349)	Redo-OPCAB (*n* = 143)	Redo-CABG (*n* = 206)	*P-*value
Age (years)	66.6 (8.5)	67.8 (8.1)	65.7 (8.6)	0.02
Female gender (%)	53 (15)	21 (15)	32 (15.5)	0.82
BMI (kg/m^2^)	27.2 (4.2)	27.9 (4.4)	26.9 (4.7)	0.07
Reduced LVEF <50%	117 (33)	46 (32)	71 (34)	0.65
Diabetes (%)	78 (22)	31 (22)	47 (23)	0.80
Hypertension (%)	252 (72)	110 (77)	142 (69)	0.08
CKD (%)	6 (2)	2 (1)	4 (2)	0.70
Preoperative creatinine (µmol/l)	103.1 (29)	101.8 (26.3)	103.9 (30.8)	0.52
eGFR (ml/min/1.73 m^2^)	64.4 (16.8)	64.7 (16.4)	64.2 (17.1)	0.76
Previous CVA (%)	40 (11)	18 (13)	22 (11)	0.56
PVD (%)	72 (21)	38 (27)	34 (16)	0.02
COPD (%)	53 (15)	25 (17)	28 (14)	0.31
Smoking history (%)	266 (76)	110 (77)	156 (76)	0.76
EuroSCORE	7 (2.7)	6.9 (2.5)	7.2 (2.8)	0.49
NYHA Class 3 or 4 (%)	149 (43)	53 (37)	96 (47)	0.10
CCS Class 3 or 4 (%)	228 (65)	89 (62)	139 (67)	0.37
Urgent surgery (%)	138 (39)	57 (40)	81 (39)	0.91
Previous surgical procedure (%)				0.03
CABG	273 (78)	100 (71)	173 (83)	
Valve	43 (12)	23 (16)	20 (10)	
CABG + valve ± other	33 (9)	18 (13)	15 (7)	
Number of diseased vessels	2.4 (0.7)	2.3 (0.8)	2.6 (0.6)	<0.01
Use of nitrates (%)	53 (15)	19 (13)	34 (16)	0.41
Number of grafts	2.5 (0.7)	2.3 (0.8)	2.6 (0.6)	<0.01
Use of IMA (%)	175 (50)	81 (57)	94 (46)	0.06
Use of radial artery (%)	67 (19)	32 (22)	35 (17)	0.26

Data are reported as mean and SD for continuous variables and as total count and percentages for categorical variables.

BMI: body mass index; LVEF: left ventricular ejection fraction; CKD: chronic kidney disease; eGFR: estimated glomerular filtration rate; CVA: cerebrovascular accident; PVD: peripheral vascular disease; COPD: chronic obstructive pulmonary disease; NYHA: New York Heart Association; CCS: Canadian Cardiovasular Society; CABG: coronary artery bypass grafting; CAD: coronary artery disease; IMA: internal mammary artery.

**Table 2:** Operative outcomes in the unmatched population

	Unmatched analysis			
	Overall (*n* = 349)	Redo-OPCAB (*n* = 143)	Redo-CABG (*n* = 206)	*P-*value
In-hospital mortality (%)	15 (4)	3 (2)	12 (6)	0.09
AKI (%)	50 (14)	18 (13)	32 (16)	0.43
CVA (%)	3 (1)	1 (1)	2 (1)	0.78
Use of IABP (%)	25 (7)	4 (3)	21 (10)	0.01
Composite outcome (%)	73 (21)	23 (16)	50 (25)	0.06
Reoperation for bleeding (%)	8 (2)	1 (1)	7 (3)	0.19
Completeness of revascularization (%)	257 (74)	108 (76)	149 (73)	0.92
Hospital stay (days)	8.6 (6.6)	8.2 (6.2)	8.8 (6.9)	0.07

Data are reported as mean and SD for continuous variables and as total count and percentages for categorical variables.

AKI: acute kidney injury (defined as peak of postoperative creatinine >% higher than preoperative value); CVA: cerebrovascular accident; IABP: intra-aortic balloon pump (either intraoperative or postoperative).

**Figure 2:**
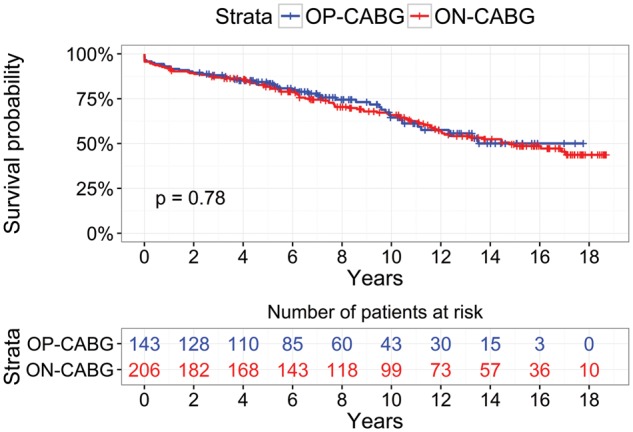
Kaplan–Meier survival curves for redo-OPCAB and redo-CABG for the entire cohort.

The mean follow-up time was 8.88 ± 5.1 years for the entire population. Figure 2 shows the Kaplan–Meier survival curves for the 2 unmatched groups. The long-term survival was similar between groups (log-rank test: *P* = 0.78). Late survival at 1-, 5- and 10-year was similar between groups at 95 vs 91.3%, 86.7 vs 83.5%, and 67.2 vs 68.4% for redo-OPCAB vs redo-CABG respectively. A multivariate logistic regression model indicated body mass index [odds ratio (OR_ 0.93, 95% confidence interval (CI) 0.86–0.99, *P* = 0.04) and EuroSCORE (OR 1.18, 95% CI 1.06–1.31, *P* = 0.01) as independent predictors of the composite outcome.

### Propensity score matching analyses A and B

Distribution of baseline characteristics for both matched analyses A and B was similar between groups (Table 3).

**Table 3:** Pre- and intraoperative characteristics of patients in matched analysis

Characteristic	Matched Analysis A				Matched Analysis B			
	Redo-OPCAB (*n* = 111)	Redo-CABG (*n* = 111)	*P*-value	SMD*	Redo-OPCAB (*n* = 88)	Redo-CABG (*n* = 88)	*P*-value	SMD*
Age (years)	67.8 (8.6)	67.9 (7.2)	0.53	0.01	67.3 (7.7)	67.7 (7.5)	0.74	0.05
Female gender (%)	15 (13)	12 (11)	0.54	0.08	11 (13)	9 (11)	0.65	0.07
BMI (kg/m^2^)	27.4 (3.7)	27.1 (4)	0.49	0.09	27.8 (3.9)	27.4 (4.3)	0.65	0.08
Reduced LVEF <50%	40 (36)	38 (34)	0.78	0.04	29 (34)	30 (36)	0.86	0.03
Diabetes (%)	26 (23)	29 (26)	0.63	0.06	19 (23)	23 (27)	0.44	0.11
Hypertension (%)	83 (75)	81 (73)	0.76	0.04	60 (71)	62 (74)	0.70	0.05
CKD (%)	1 (1)	1 (1)	1.00	<0.01	0 (0)	1 (1)	NA	0.16
Preop creatinine (µmol/l)	100.1 (25.4)	102 (29)	0.76	0.07	96.7 (20)	98.6 (24)	0.82	0.08
eGFR (ml/min/1.73 m^2^)	66 (16)	65.8 (18)	0.84	0.01	67.9 (16)	67.7 (17.7)	0.83	0.01
Previous CVA (%)	15 (13)	14 (13)	0.84	0.03	9 (11)	9 (11)	1.00	<0.01
PVD (%)	23 (21)	25 (22)	0.73	0.04	13 (15)	15 (18)	0.66	0.06
COPD (%)	20 (18)	17 (15)	0.59	0.07	10 (12)	8 (9)	0.59	0.08
Smoking history (%)	82 (73)	81 (73)	0.88	0.02	65 (78)	60 (71)	0.35	0.14
EuroSCORE	7 (2.7)	6.9 (2.7)	0.83	0.05	7.1 (2.5)	7.2 (2.8)	0.71	0.06
NYHA Class 3/4 (%)	43 (39)	44 (40)	0.89	0.02	35 (42)	33 (39)	0.76	0.05
CCS Class 3/4 (%)	67 (60)	73 (66)	0.43	0.11	57 (68)	55 (65)	0.73	0.05
Urgent surgery (%)	44 (40)	48 (44)	0.57	0.07	33 (39)	28 (33)	0.41	0.12
Previous type of surgery (%)			0.91	0.06				
CABG	84 (76)	83 (75)			NA	NA		
Valve	16 (14)	18 (16)			NA	NA		
CABG + valve ± other	11 (10)	10 (9)			NA	NA		
Number of diseased vessels	2.4 (0.7)	2.4 (0.7)	0.79	0.03	2.3 (0.8)	2.4 (0.7)	0.29	0.13
Use of nitrates (%)	18 (16)	17 (15)	0.84	0.03	11 (13)	10 (12)	0.78	0.04
No of grafts	2.1 (0.8)	2.4 (0.8)	<0.01		2 (0.8)	2.2 (0.8)	0.03	
Use of IMA (%)	62 (56)	52 (47)	0.21		44 (51)	29 (34)	0.02	
Use of radial artery (%)	24 (22)	19 (17)	0.39		20 (24)	16 (19)	0.47	

Data are reported as mean and SD for continuous variables and as total count and percentages for categorical variable.

SMD: standardized mean difference (reported for the variables included in the propensity score matching process); BMI: body mass index; LVEF: Left ventricular ejection fraction; CKD: chronic kidney disease; CVA: cerebrovascular accident; PVD: peripheral vascular disease; COPD: chronic obstructive pulmonary disease; NYHA: New York Heart Association; CCS: Canadian Cardiovascular Society.

a SMD (reported for the variables included in the propensity score-matching process).

#### Matched Analysis A

**Figure 3:**
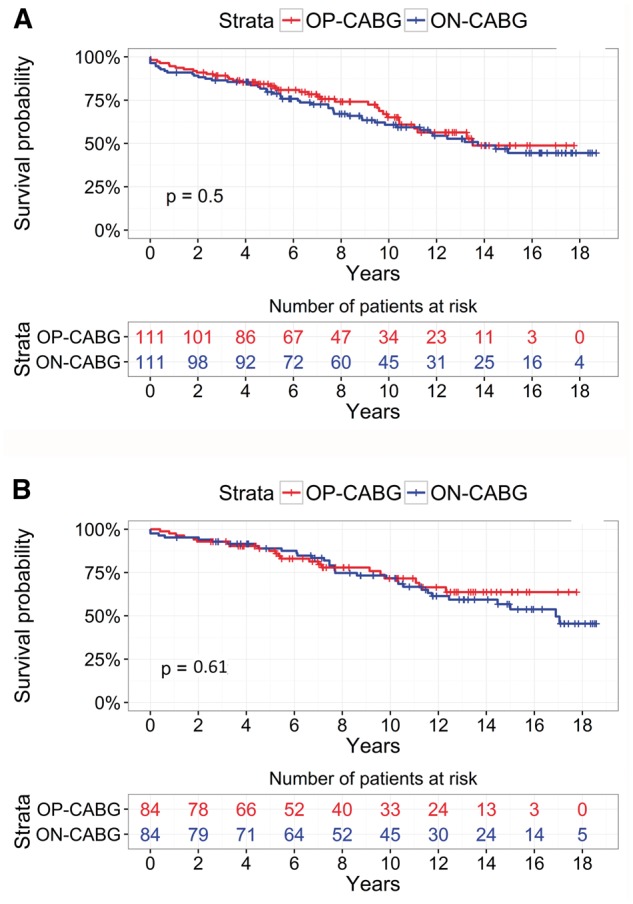
Kaplan–Meier survival curves for redo-OPCAB and redo-CABG in the propensity score-Matched Analysis A and Matched Analysis B.

In propensity score-Matched Analysis A the number of patients was 111 in each group, including those who had undergone any type of primary surgery (CABG only, valve only and CABG plus valve with/without other). Postoperative outcomes are shown in Table 4. The rate of the composite end-point was 21% in the redo-CABG and 17% in the redo-OPCAB groups (*P* = 0.25). This included in-hospital mortality (4 vs 2%, *P* = 0.25), AKI (14% vs 12%, *P* = 0.56), stroke (1% vs 1%, *P* = 1) and severe LCO requiring IABP (10 vs 3%, *P* = 0.03), all redo-CABG vs redo-OPCAB. Reopening for bleeding was 4 times higher in the redo-CABG group (4% vs 1%, *P* = 0.17) and the length of stay was slightly longer in the redo-CABG group (8.7 ± 5.6 vs 8.1 ± 5.6 days, *P* = 0.18). Peak of postoperative SCrea was 127.9 ± 59.8 µmol/l and 123.3 ± 60.5 µmol/l for redo-CABG and redo-OPCAB, respectively (*P* = 0.34). Figure 3A shows the Kaplan–Meier survival curves. The long-term survival was similar between groups (log-rank test: *P* = 0.5). Late survival at 1, 5 and 10 years was similar between groups at 94.6 vs 91%, 83.2 vs 79.8% and 65.1 vs 60.8% for redo-OPCAB vs redo-CABG, respectively. The number of graft was higher in the redo-CABG group (2.4 ± 0.8 vs 2.1 ± 0.8, *P* <0.01). After stepwise selection process, the multivariable logistic regression model included EuroSCORE (OR = 1.25, 95% CI: 0.99–1.59, *P* = 0.06) and preoperative creatinine (OR = 1.02, 95% CI 0.99–1.04, *P* = 0.15) as predictors of composite outcome.

**Table 4:** Operative outcomes in matched analysis

	Matched Analysis A			Matched Analysis B		
Characteristic	Redo-OPCAB (*n* = 111)	Redo-CABG (*n* = 111)	*P*-value	Redo-OPCAB (*n* = 84)	Redo-CABG (*n* = 84)	*P*-value
In-hospital mortality (%)	2 (2)	5 (4)	0.25	0 (0.0)	4 (5)	0.13
AKI (%)	13 (12)	16 (14)	0.56	5 (6)	9 (11)	0.24
CVA (%)	1 (1)	1 (1)	1.00	0 (0)	0 (0)	NA
Usage of IABP (%)	3 (3)	10 (10)	0.03	2 (2)	9 (11)	0.02
Composite outcome (%)	17 (15)	23 (21)	0.25	7 (8)	16 (19)	0.04
Reoperation for bleeding (%)	1 (1)	4 (4)	0.17	0 (0)	1 (1)	1.00
Completeness of revascularization (%)	77 (69)	91 (82)	0.02	57 (68)	60 (71)	0.60
Hospital stay	8.1 (6.2)	8.67 (5.6)	0.15	8 (5.4)	8.5 (5)	0.17

AKI: acute kidney injury (defined as peak of postoperative creatinine >% higher than preoperative value); CVA: cerebrovascular accident; IABP: intra-aortic balloon pump (either intraoperatively or postoperatively only).

#### Matched Analysis B

In propensity score-Matched Analysis B, the number of patients was 84 in each group, including those undergone isolated CABG only as the primary operation. Postoperative outcomes are shown in Table 4. The rate of the composite end-point was reduced in the redo-OPCAB group (19 vs 8%, *P* = 0.04) and distributed as follows: in-hospital mortality (5 vs 0%, *P* = 0.13), AKI (11 vs 6%, *P* = 0.24), stroke (0% vs 0.0%, *P* = NA) and severe LCO requiring IABP (11 vs 2%, *P* = 0.02), all redo-CABG vs redo-OPCAB, respectively. Reopening for bleeding was 1% vs 0.0% (*P* = 1), completeness of revascularization was 71 vs 68% (*P* = 0.6) and length of stay was 8.5 ± 5.4 vs 8.5 ± 5 days (*P* = 0.17), all redo-CABG vs redo-OPCAB, respectively. Peak of postoperative SCrea was 135.3 ± 100.6 µmol/l and 110 ± 45 µmol/l for redo-CABG and redo-OPCAB, respectively (*P* = 0.01). Figure 3B shows the Kaplan–Meier survival for the 2 groups. The long-term survival was similar between groups (log-rank test: *P* = 0.61). Late survival at 1, 5, and 10 years was 97.6 vs 95.2%, 87.5 vs 88.9% and 71.6 vs 71.7% for redo-OPCAB vs redo-CABG, respectively. The multivariable logistic regression model identified redo-CABG (OR = 3.83, 95% CI: 1.14–12.8, *P* = 0.03) and urgent surgery (OR = 9.8, 95% CI 0.91–104.2, *P* = 0.06) as predictors for composite outcome.

## DISCUSSION

Only few studies have reported on the use of OPCAB technique for redo coronary surgery [1, 6, 16–19]. The largest report in 617 patients from Japan focuses on in-hospital outcome only with no late survival and suggests that redo-OPCAB surgery is associated with lower 30 days mortality (3.5 vs 7%) and less complications (11 vs 21.5%) compared to redo-CABG [1]. Other studies from North America and India have reported on redo-OPCAB, although in small cohorts [1, 7, 17, 18, 20, 21].

Our unmatched analysis suggests that redo-OPCAB is associated with reduced composite end-point and similar 10-year survival when compared with redo-CABG. In this analysis, the effect size of redo-OPCAB appears marked for mortality and need for IABP that were 3 times more common in the redo-CABG group. Our propensity score-Matched Analysis A (any cardiac procedure as primary operation) showed no differences in early composite end-point (21 vs 15%) and 10-year survival (65.1 vs 60.8%) between redo-CABG and redo-OPCAB, respectively. Conversely, the propensity score-Matched Analysis B (isolated coronary surgery as primary operation) showed a reduction in the early composite end-point by >50% (19 vs 8%), with marked difference in mortality (5 times lower—5 vs 0%, *P* = 0.13) and severe LCO needing IABP (4.5 times lower—11 vs 2%, *P* = 0.02), both favouring redo-OPCAB; 10-year survival was similar to redo-CABG (71.6% vs 71.7%), respectively.

The 30-day mortality for redo-OPCAB in Matched Analysis B was lower than that reported in the Japanese study (1.1% vs 3.5%). This difference could reflect differences in risk profile. However, in both studies redo-OPCAB was associated with lower mortality than redo-CABG. This is confirmed by others. Sabik *et al.* reported the outcome of 4518 redo-CABG reoperations [2] with mortality at 4.3% for first redo, 5.1% for second redo and 6.4% for third redo or more. Similar results were reported by Ghanta *et al.* in 72 322 redo-CABG procedures from the STS database [3].

Our study and the report by Dohi *et al.* [1] also suggest less postoperative complications following redo-OPCAB. Others have reported more complications following redo-OPCAB than our study, although still less than the rate observed for the redo-CABG groups. Morris *et al.* [22] reported 41.3% of postoperative complications after redo-CABG vs 25% after redo-OPCAB (*P* <0.01), in keeping with the outcome of a small UK study [23].

Shin and colleagues [18] reported postoperative complication rates of 64.2% vs 33.3% (*P* = 0.08) between redo-CABG and redo-OPCAB, respectively. These findings could explain a tendency for the shorter hospital stay observed with redo-OPCAB in our study, which was 0.6, 0.75 and 1.3 days shorter for the unmatched, Matched B and Matched A analyses, respectively. This is in keeping with the reports by others [1, 18, 20, 22].

The two propensity score-matched analyses of this study suggested that redo-OPCAB may be more effective when used for redo patients who have undergone isolated coronary surgery as their primary operation. This is highlighted by the rates of early composite end-point, which was 8% vs 15% for the redo-OPCAB groups for Matched Analysis B vs Matched Analysis A, respectively (Table 4). This finding was not observed for redo-CABG cohorts when using a similar comparative approach.

An important finding of the current study is that in terms of the 10-year survival redo-OPCAB is as effective as conventional redo-CABG across all the 3 analyses undertaken. This finding is reassuring when considering the technical complexity involved in undertaking coronary anastomoses on the beating heart within the context of a redo cardiac procedure. This finding is in keeping with the long-term outcome of our previous BHACAS trial reporting on long-term graft patency and survival following primary OPCAB surgery [14].

Our propensity score-Matched Analysis B showed similar 1- and 5-year survival between groups at 97.6 vs 95.2% and 87.5 vs 88.9% for redo-OPCAB vs redo-CABG, respectively. This finding is similar or better than that reported by others following redo coronary surgery. Usta *et al.* [19] reported in a small study a 3-year survival rate of 81 ± 12vs 63 ± 9% in redo-OPCAB vs redo-CABG. Tugtekin *et al.* [21] reported in another small study a 3-year survival rate of 83.8 vs 88.6% for redo-CABG vs redo-OPCAB. In a further small study with 43 patients in each group, actuarial survival at 5-year was 87 ± 5.5% for redo-CABG and 95 ± 3.2% for redo-OPCAB (*P* = 0.17) [23].

Completeness of coronary revascularization has been associated with long-term outcome [17, 21, 24]. Some evidence suggests that this may be a limit for OPCAB surgery [25], with a retrospective analyse suggesting that OPCAB is an independent predictor of incomplete revascularization [17, 20, 21, 24]. Our study showed similar completeness of revascularization between groups in the unmatched analysis but better results in the redo-CABG group in Matched Analysis A. In Matched Analysis B, completeness of revascularization favoured only slightly redo-CABG surgery (71 vs 68%; *P* = 0.60), although the number of grafts needed was reduced in the redo-OPCAB group (21 ± 0.8 vs 2.4 ± 0.8; *P* = 0.03) reflecting the baseline difference in number of diseased vessels. This finding might reflect a tendency to perform redo-OPCAB in patients with reduced number of diseased coronaries as suggested by others [22], but this speculation cannot be confirmed in our study.

The ESC/EACTS guidelines suggest that in view of the higher risk of procedural mortality with redo-CABG and the similar long-term outcome, percutaneous coronary intervention is the preferred revascularization strategy in CABG patients requiring redo revascularization [26]. The findings of our Matched Analysis B suggests an overall in-hospital mortality of 2.5% (0% for redo-OPCAB) and 1-, 5-, and 10-year survival rates of 96, 88 and 71.6%, respectively. These remarkable results question the rationale of treating these patients with percutaneous coronary intervention stenting [5] and call for a more in-depth evaluation of the available evidence.

### Limitations

There are several limitations to this study. It is a retrospective single-centre analysis in a limited cohort. The allocation of patients to the study group was by surgeon’s expertise, and this might have led to undetected difference in risk profile between groups. The study is from an Institution with historical high interest and expertise in OPCAB surgery, and this might limit the applicability of the findings to Institutions with less interest and proficiency in OPCAB surgery. The study included a patient cohort treated over a long time period, hence with possible confounding factors due to changes in clinical practice overtime. Finally, the evaluation of long-term impact of redo-OPCAB surgery was limited to all-cause mortality as no data were available on cardiac-related mortality or late graft patency.

## CONCLUSION

In conclusion, this study suggests that redo coronary surgery in general can be undertaken at low early mortality and at high 1-, 5-, and 10-year survival. It also shows that redo-OPCAB surgery is feasible and safe in this complex surgical scenario with better early in-hospital outcome compared to conventional redo-CABG and comparable high 10-year survival.

## ACKNOWLEDGEMENTS

The authors would like to thank A. Davis, Clinical Audit Officer of the Bristol Heart Institute.

## Funding 

This study was supported by the National Institute for Health Research Biomedical Research Unit in Cardiovascular Disease. Ascione is supported by research grants from the British Heart Foundation and the Medical Research Council.

**Conflict of interest:** none declared.
